# The Prospective Regulatory Functions of lncRNAs and Their ceRNA Networks in the Development of Motor Neurons and Associated Diseases

**DOI:** 10.3390/biom16081208

**Published:** 2026-08-19

**Authors:** Zhenzhen Wang, Yuhan Fu, Siqi Li, Yan Zhang, Tao Sun, Nan Miao

**Affiliations:** Center for Precision Medicine, School of Medicine and School of Biomedical Sciences, Huaqiao University Xiamen Campus, No. 668 Jimei Street, Xiamen 361021, China; 23014071016@stu.hqu.edu.cn (Z.W.); 24014071007@stu.hqu.edu.cn (Y.F.); 23013071016@stu.hqu.edu.cn (S.L.); 25013071022@stu.hqu.edu.cn (Y.Z.); taosun@hqu.edu.cn (T.S.)

**Keywords:** MND, lncRNA, ceRNA network, ALS, SMA, MS

## Abstract

Motor neurons form a highly specialized network composed of α-, β-, and γ-subtypes that coordinate skeletal muscle activity. Motor neuron diseases (MNDs), including amyotrophic lateral sclerosis (ALS) and spinal muscular atrophy (SMA), are characterized by the progressive degeneration of this network, resulting in motor dysfunction. Emerging evidence underscores the significant roles of long non-coding RNAs (lncRNAs) in motor neuron development and disease. However, only a few have been experimentally confirmed as true ceRNA regulators, highlighting the need to differentiate validated mechanisms from mere associations or predictions. This review summarizes the regulatory roles of lncRNA-associated ceRNA networks in motor neuron development, evaluates the evidence for their involvement in MNDs, and explores their potential impact on disease progression. It also addresses current challenges, knowledge gaps, and future research directions for understanding ceRNA-mediated mechanisms and developing therapeutic strategies for MNDs.

## 1. Introduction

Alpha (α) and gamma (γ) MNs are responsible for conveying neural signals from the brain and spinal cord to the muscles [1,2]. Abnormalities in these neurons can result in MNDs, including ALS, which is characterized by protein aggregation [3,4,5], and SMA, which involves a deficiency of survival motor neuron (SMN) protein in anterior horn cells [6]. LncRNAs are implicated in motor neuron development and diseases such as ALS and SMA across various species [7,8]. For example, lncRNA *Lhx1os* regulates the postnatal reduction of mature MNs [9], *C9ORF72-AS* contributes to toxic dipeptide repeat production [10], and *SMN-AS1* represses *SMN1* transcription in SMA patients and mice [11]. These alterations exacerbate neuroinflammation and disrupt proteostasis, hastening neuronal damage. Although lncRNAs are typically considered non-abundant, advanced next-generation sequencing techniques have revealed that certain lncRNAs are expressed at detectable levels in specific cell types, particularly in MNs [12,13,14].

Competing endogenous RNAs (ceRNAs) represent a class of RNAs characterized by the presence of similar, if not identical, microRNA (miRNA)-responsive elements (MREs) for shared miRNAs, thereby facilitating mutual regulation through competitive miRNA binding [15,16,17]. To advance the study of ceRNA networks, various computational tools and databases have been developed. For instance, the LnCeVar database provides a comprehensive resource for genomic variations that disrupt ceRNA network regulation based on single-cell/spatial transcriptomics data [18,19]. Additionally, platforms such as CeRNASeek and Crinet enable the identification and analysis of ceRNA interactions by integrating multi-omics data, thereby enhancing the understanding of ceRNA regulation in complex diseases [20,21].

As post-transcriptional regulators, ceRNAs are implicated in diverse biological processes and play a significant role in motor neuron development and associated diseases [22]. Examples include *CyCoNP*/*miR-4492*/*NCAM1* in Human Induced Pluripotent Stem Cells (hiPSC)-derived MN [23], and *miR-34a*/*Spag5*/*lncRNA00138536-circRNA007386* in SMA mice model [24]. Despite the growing number of disease-associated lncRNAs reported in recent years, only a limited number have been experimentally validated as bona fide ceRNA regulators. Most lncRNA–miRNA–mRNA interactions are based on correlations, patient data, or predictions rather than thorough functional validation, underscoring the need for more rigorous evaluation of lncRNA-mediated ceRNA regulation in motor neuron development and associated diseases.

This review summarizes the roles of lncRNAs in motor neuron development, focusing on their ceRNA-related regulatory mechanisms. It examines evidence for lncRNA-mediated ceRNA networks in major motor neuron diseases like ALS and SMA, and their relevance in non-motor-neuron diseases like MS. The review evaluates the evidence strength, differentiates between experimentally validated mechanisms and predictions, and discusses limitations, therapeutic prospects, and future research directions, aiming to offer a comprehensive understanding of lncRNA-mediated ceRNA regulation in motor neuron biology and disease.

## 2. Motor Neuron Development and Related Diseases

MNs connect the central nervous system (CNS) to skeletal muscles, enabling voluntary movement through processes like cell fate determination, axon growth, target innervation, and synapse formation [25,26,27,28]. Understanding these mechanisms is essential for elucidating neural circuit formation and the pathogenesis of related neurodegenerative diseases.

### 2.1. Motor Neuron Development and Columns

MNs are generated through the coordinated actions of multiple signaling pathways (Shh, BMP, Wnt etc.) in neural progenitors along the spinal cord’s dorsoventral axis during embryogenesis [29,30]. They originate from the *Olig2*-expressing motor neuron progenitors (pMN) domain in the ventral neural tube and form distinct motor columns along the rostrocaudal axis, determined by *Hox* gene expression patterns [25,26,31,32]. MNs are classified into upper MNs (UMNs) in the motor cortex, and lower MNs (LMNs) in the brainstem and spinal cord [27]. UMNs connect to LMNs and control voluntary movement, and damage to UMNs results in spasticity, hyperreflexia, and impaired fine motor skills [28]. LMNs directly innervate muscles or glands and their damage leads to flaccid paralysis, muscle atrophy, and loss of reflexes. LMNs include branchial MNs for cranial muscles, visceral MNs for smooth muscles and glands, and somatic MNs for skeletal muscles.

In the vertebrate spinal cord, MNs are organized into distinct columns, including the medial motor column (MMC) for axial muscles, the hypaxial motor column (HMC) for body wall muscles, and the lateral motor column (LMC) for limb musculature (Table 1) [33,34,35,36].

#### 2.1.1. MMC

MMC neurons are early-born motor neurons that extend along the spinal cord, control posture and trunk stability by innervating axial muscles, initiated in the pMN domain under the influence of Shh signaling and is considered largely independent of *Hox* gene patterning at early stages (Table 1). A dorsal-ventral Wnt4/5 signaling gradient supports MMC fate by maintaining Lhx3/4 protein expression, and its loss impairs MMC generation, while increased activity enhances it [37]. FGF9 acts as a survival factor for thoracic and sacral MMC neurons, while contributes to the organization of MMC and LMC neurons into motor pools [38,39]. In vitro, Shh agonists and retinoic acid (RA) can generate MMC-like neurons from stem cells, characterized by Lhx3 expression and medial positioning with epaxial muscle targeting [46,58]. MN function is further shaped by inhibitory spinal interneurons, and early loss of inhibitory V1 interneuron synaptic inputs onto motoneurons has been implicated in the early pathogenesis of amyotrophic lateral sclerosis (ALS).

#### 2.1.2. LMC

LMC neurons innervate limb muscles, with medial LMC (LMCm) neurons projecting to ventral limb musculature and lateral LMC (LMCl) neurons projecting to dorsal limb muscles [47,59]. Neuroanatomical tracing reveals diverse LMC functions and connections, identifying specific motor pools and subpopulations of α- and γ-MNs [47]. EphrinB2 regulates LMC boundary and axon pathfinding [47], and alterations in the mesenchymal environment of conditional *Bmpr1a* mutants result in the misrouting of LMCm axons [30]. As well, RA metabolism influenced by *Cyp26a1*, affects LMC specification with *Hoxc6* up regulation [49]. Markers like SC1 aid in neuron adhesion and axon organization [33]. In ALS *SOD1^G93A^* mice, LMC degeneration is linked to a decrease in the *miR-17~92* nuclear PTEN axis [50]. These findings highlight that LMC identity is influenced by both intrinsic transcriptional programs and varied connectivity.

#### 2.1.3. HMC

HMC is key for forming sensorimotor circuits needed for coordinated movement and originate from *Olig2^+^* progenitors and lack FoxP1 [51,52,54]. It is also influenced by *Kdm6b* in thoracic segments for breathing and trunk stability, which collaborates with the ISL1-LHX3 protein complex to promote HMC and MMC fates and inhibit LMC identities [53,56]. Disruptions in HMC can affect motor neuron identity and connectivity, potentially leading to respiratory dysfunction in diseases such as SMA and ALS and may increase the vulnerability of specific motor neuron subtypes in adulthood.

#### 2.1.4. Subtype-Specific lncRNA Expression in MMC, LMC and HMC

ScRNA-seq and scStereo RNA-seq are powerful techniques for generating RNA transcript profiles at the individual cell level, particularly for lncRNAs, which are often underrepresented in bulk RNA sequencing due to their low abundance. Recent studies conducted by our research group have demonstrated that lncRNAs such as *BASP-AS1/ISL1-DT* were highly expressed in the MMC of human fetus spinal cord, *SNHG14/LINC00632* in the HMC, and *LINC02381/FOXD3-AS1* in the LMC [14]. Furthermore, several *cis* gene pairs, including *ISL1-DT/ISL1* (MMC), *MEG3/RTL1* (HMC), and *FOXD3-AS1/FOXD3* (LMC), have been validated using similar spinal scStereo RNA-seq slides [14]. Nonetheless, the precise regulatory roles of these lncRNAs remain to be elucidated.

### 2.2. Somatic Motor Neurons (sMNs) and Related Diseases

MN subtypes, including α, β, and γ-MNs, exhibit functional diversity crucial for precise motor tasks [60,61,62]. This diversity is characterized by their unique muscle targets and distinct transcriptional profiles, such as receptor ERR3 and ESRRG, which is enriched in γ-MNs, whereas α-MNs are typically protein NeuN-positive. In this section, we introduce three MN subtypes and related diseases (Table 2).

#### 2.2.1. α-MNs and Related Diseases

α-MNs control voluntary and reflexive skeletal muscle movements and interact with V2a interneurons in zebrafish to generate locomotor rhythm [76]. They are larger than γ-MNs, have distinct firing patterns, and remain resilient with age despite reduced synaptic inputs, contributing to age-related motor issues [65]. Hyperpolarization-activated cyclic nucleotide-gated channel 1 (HCN1) channels contribute to the regulation of neuronal excitability [77], disrupted HCN1 pathways via VAPB mutations may lead to α-MN vulnerability in ALS and SMA [65,76,77]. In the SOD1^G93A^ mouse model of ALS, misfolded SOD1 triggers cytosolic phospholipase A2 alpha (cPLA2α) in MNs [78], while SMA involves reduced SMN protein levels with *SMN1* mutations, causing α-MN loss and muscle atrophy [65] (Table 2).

ALS is marked by disrupted proteostasis, with over 95% of cases showing TAR DNA-binding protein 43 (TDP-43) mis-localization [79,80]. Mutations in SOD1, FUS, as well as dipeptide repeat proteins (DPR) from C9ORF72 expansions also lead to proteostasis issues [81,82,83], contributing to α-MNs degeneration through mechanisms like ER stress and impaired autophagy [82,84,85,86,87].

SMA is classified into types 0–IV based on onset and motor function. In SMA, α-MNs are significantly reduced due to SMN protein deficiency affecting RNA metabolism and splicing, while γ-MNs and other cholinergic neurons remain mostly unaffected [65]. Recent treatments focus on increasing SMN protein levels, and epidural spinal cord stimulation has been shown to enhance strength, endurance, and gait by boosting α-MNs firing rates [88,89].

Although Multiple sclerosis (MS) is not usually classified as a motor neuron disease, evidence suggests that α-motor neuron dysfunction contributes to motor impairment. Recent studies reveal altered motor unit firing, imbalanced excitatory-inhibitory signals, decreased discharge rates, reduced torque steadiness, and increased fatigue in MS patients [68]. These findings suggest that α-motor neuron output abnormalities may contribute to the varied motor deficits in MS.

Collectively, these findings indicate that α-MNs are highly susceptible to diverse pathological insults, including proteostasis dysfunction, RNA-processing defects, and impaired neural drive, making them a central target in several motor-related neurological disorders.

#### 2.2.2. β-MNs and PLS

β-MNs innervate both extrafusal and intrafusal fibers, coordinating muscle contraction with proprioceptive length feedback. Cortical beta-band oscillations (13–30 Hz) are transmitted via fast-conducting corticospinal pathways to spinal motor neuron pools, modulating overall motor output and serving as potential biomarkers for neurological conditions [90]. For instance, β-band intermuscular coherence is essential for UMN dysfunction in PLS, relying on an intact corticospinal tract and persisting even with anterior horn cell damage [69]. The degeneration of β-MNs in PLS is associated with oxidative stress and lipid peroxidation (Table 2).

PLS, differs from ALS by exhibiting notable atrophy in the primary motor cortex and corticospinal tracts, with minimal LMN involvement, as shown by fewer ubiquitin and TDP-43 inclusions [71]. TMS studies indicate high cortical thresholds and delayed conduction times, pointing to UMN dysfunction in PLS [91].

While research on β-MNs is limited, evidence indicates their dual innervation pattern may play a role in motor dysfunction related to upper motor neuron disorders, warranting further study.

#### 2.2.3. γ-MNs and ALS

γ-MNs control spindle tension and sensory sensitivity by innervating intrafusal fibers within muscle spindles, which can improve motor command accuracy [72]. Computational models suggest that advancing the input phase to dynamic γ-MNs compared to α-MNs can enhance low-frequency components of synaptic input, boosting the control-to-neural-noise ratio during movement [92]. Additionally, the orphan nuclear receptors ERR2/3 are crucial for developing the γ-MN properties in proprioceptive movement control [73,93] (Table 2).

Developmental differences between α-MNs and γ-MNs are marked by *Wnt7a* expression during embryonic development, continuing postnatally [74]. The absence of glial cell line-derived neurotrophic factor (GDNF) specifically leads to the γ-MNs loss but not the muscle spindle or its sensory innervation. In ALS, γ-MNs are impacted by cortical hyperexcitability, indicating early cortical dysfunction as a potential target [94]. Unlike α-MNs, γ-MNs show resistance to degeneration in various ALS models, with fast-fatigable α-MNs being most vulnerable [72,95]. Surviving γ-MNs may increase proprioceptive input to vulnerable α-MNs, influencing circuit remodeling and disease progression [96], highlighting both cell-autonomous and non-cell-autonomous factors in motor neuron vulnerability in ALS.

Overall, γ-MNs uniquely resist degeneration in ALS and may indirectly impact disease progression through changes in proprioceptive circuitry.

#### 2.2.4. Subtype-Specific lncRNA Expression in α-, β-, and γ-Motor Neurons

Recent advances have reliably identified α- and γ-motor neurons and a putative γ* subpopulation, while β-motor neurons remain difficult to distinguish owing to their intermediate transcriptional characteristics [97]. Some lncRNAs are known to play roles in motor neuron development. For example, *Meg3* regulates Hox gene expression and maintains epigenetic marks during motor neuron differentiation [14,97]. In the MN precursors, *HOTAIRM1* are highly expressed in VZ and SVZ in ventral fetus spinal cord, while *HOXA-AS2* are broadly expressed in both dorsal and ventral domains [14]. However, whether its expression differs among α-, β-, and γ-motor neuron subtypes has not yet been determined.

## 3. LncRNA in Motor Neuron Development

**Figure 1:**
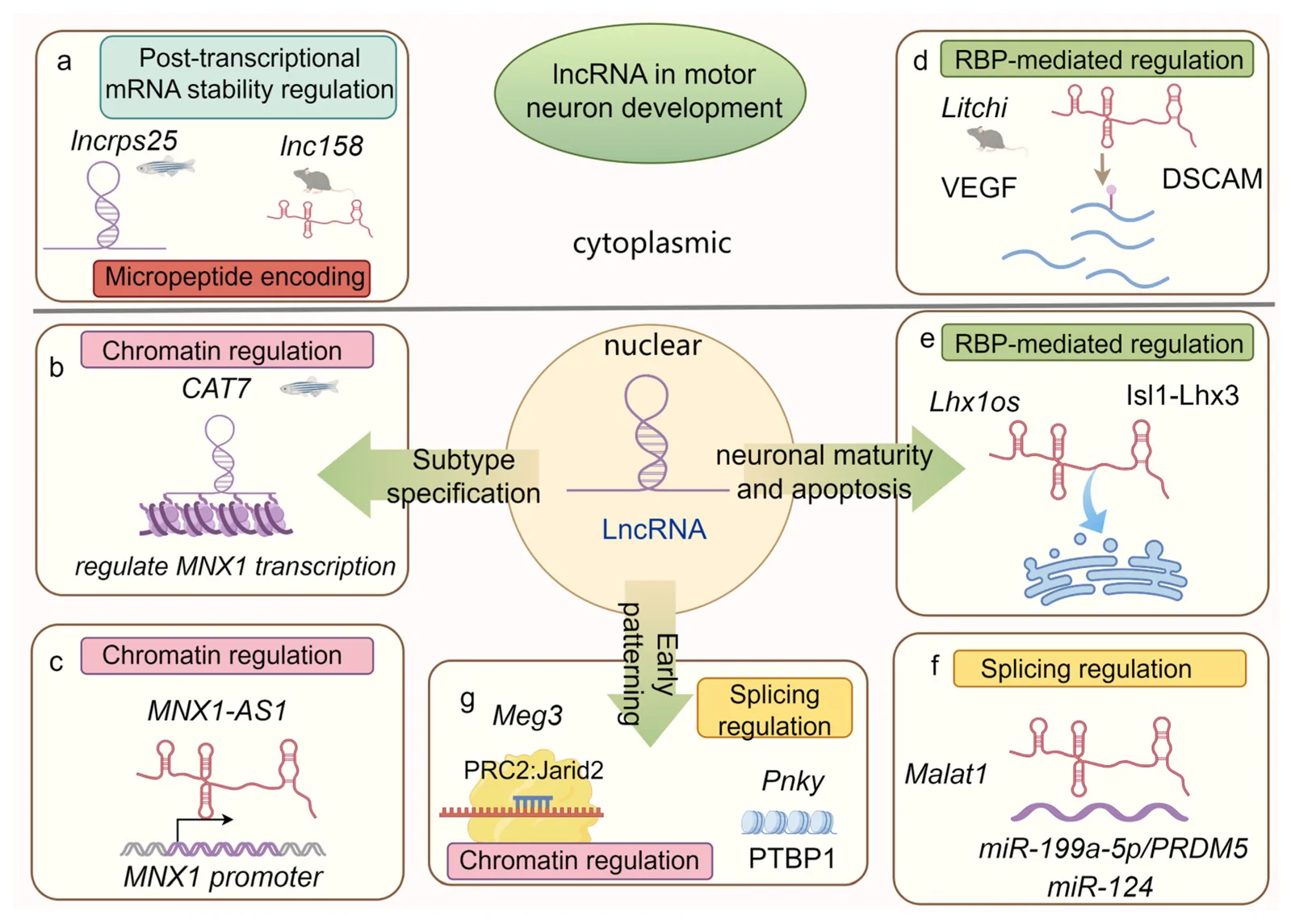
Key lncRNAs Involved in Motor Neuron Development. (**a**) *lncrps25* promotes MN formation by encoding micropeptide and regulating *Olig2*; *lnc158* regulates oligodendrocyte differentiation via NFIB. (**b**) *CAT7* activates early MN subtype identity through *MNX1* and chromatin remodeling. (**c**) *MNX1-AS1* specifies MN subtypes via *MNX1* regulation. (**d**) *Litchi* orchestrates spinal circuit assembly via DSCAM-VEGF signaling. (**e**) *Lhx1os* maintains MN homeostasis via Isl1-Lhx3 interaction. (**f**) *Malat1* controls synaptic organization and apoptosis via *miR-199a-5p*/*PRDM5* and *miR-124*. (**g**) *Meg3* establishes rostrocaudal identity via PRC2:Jarid2; *Pnky* regulates NSC migration and differentiation via *PTBP1*.

Non-coding RNAs, crucial for post-transcriptional regulation, are divided by length into lncRNAs (over 200 nucleotides) and small non-coding RNAs (under 200 nucleotides), like miRNAs [98]. LncRNAs, transcribed by RNA polymerase II, include various types based on their genomic position and mechanisms that depend on subcellular localization [7,8,15]. In the nucleus, they affect chromatin and transcription, while in the cytoplasm, they influence mRNA stability and translation [99]. Within the ceRNA network, lncRNAs act as sponges for miRNAs, impacting gene expression by reducing miRNA repression of target mRNAs [15] (Figure 1).

In this section, we summarize the regulatory roles of lncRNAs in MN development, focusing on their functions in neural patterning, subtype specification, and neuronal maturity and apoptosis.

### 3.1. Early Patterning

*Meg3*, a maternally expressed lncRNA derived from the *Dlk1-Dio3* locus, is upregulated during embryonic stem cell differentiation into MNs, especially in rostral MN subtypes [100]. Induced by RA and MN-related transcription factors, *Meg3* predominantly localizes to the nucleus, where it represses progenitor-associated and caudal *Hox* genes [100,101,102]. *Meg3* acts as a molecular scaffold to facilitate the interaction between Ezh2 and Jarid2, thereby maintaining the H3K27me3 epigenetic landscape required for preserving MN subtype identity following cell cycle exit [100] (Figure 1 and Table 3).

*Pnky*, a conserved lncRNA that forms a functional complex with the RNA-binding protein (RBP) polypyrimidine tract-binding protein (PTBP1) to regulate RNA processing and neuronal differentiation in neural stem cells (NSCs) [103,104,105]. *Pnky* directly interacts with splicing factors such as U2AF1 and SARNP, modulating the splicing and nuclear export of target mRNAs, including genes involved in cell migration (e.g., MMP2 and Paxillin), thereby promoting NSC migratory capacity [45,104]. Consistently, depletion of either *Pnky* or *PTBP1* markedly enhances neuronal differentiation of embryonic and postnatal NSC [45,105]. Emerging evidence further suggests that *Pnky* modulates NSC proliferation and differentiation through the Wnt/β-catenin signaling pathway in the context of neurodegenerative diseases such as ALS and SMA, highlighting its potential as a therapeutic target [106].

### 3.2. Subtype Specification

*MNX1-AS1*, an antisense lncRNA transcribed from the *MNX1* locus, has been implicated in motor neuron-related pathophysiological processes, potentially through its involvement in RNA processing and axonal transport [107]. In contrast, the protein-coding gene *MNX1* plays a conserved role in motor neuron subtype specification; in zebrafish, *Mnx1* is essential for the differentiation of specific motoneuron subtypes, such as MiP MNs, by maintaining subtype identity and suppressing interneuron gene expression programs [127] (Figure 1 and Table 3).

*CAT7* enhances polycomb repressive complex 1 (PRC1) binding to the *MNX1* promoter, thereby delaying premature *MNX1* activation during motor neuron subtype specification [108,109,110]. In vivo studies of zebrafish embryos further confirm a central role of PRC1 in motor neuron fate determination, as loss of the PRC1 core component *Ring1* disrupts motor neuron identity and induces ectopic expression of alternative fate determinants, including *Hox* genes [108]. This regulatory mechanism is evolutionarily conserved, as loss of *cat7l* in zebrafish leads to locomotor defects that can be rescued by human *CAT7* expression [108]. Together, these findings demonstrate that *CAT7* regulates motor neuron subtype specification by recruiting PRC1 to lineage-specific loci [111].

*lncrps25*, broadly expressed in the developing spinal cord, is required for proper MN formation and locomotor activity in zebrafish, and encoding a micropeptide crucial for vertebrate brain cell development and affecting NMDA receptor pathways [112]. It regulates influencing motor neuron and oligodendrocyte precursor cell fate, with Olig2 overexpression partially correcting related defects [113]. Reduced *lncrps25* disrupts oligodendrocyte-specific lncRNA lncOL1 and lowers Olig2 expression, leading to myelination issues and increased neuronal hyperexcitability [128,129].

*lnc158*, an endogenous antisense transcript, enhances nuclear factor-IB (NFIB) by stabilizing its mRNA via 3′UTR complementarity, which boosts myelin-related gene expression and promotes oligodendrocyte differentiation [114].

Subtype-specific lncRNAs, such as *Gm12688* in V1 interneuron and *Gm14204* in V2b interneuron are correlated with lineage determinants *Foxd3* and *Slc32a1* [130], suggesting *cis*-regulatory roles in neuronal diversification.

### 3.3. Neuronal Maturity and Apoptosis

*Malat1* is a conserved molecule that influences synaptic function and neuronal health by moving to the cytoplasm to affect local translation [120,131]. It promotes neuronal apoptosis in spinal cord injury via the *miR-199a-5p*/*PRDM5* axis and *miR-124* [122,123]. *Malat1* deficiency disrupts synaptic organization in mice, while its overexpression *Malat1* aids neural recovery by reducing microglial M1 polarization and neuroinflammation [124] (Figure 1 and Table 3).

*Litchi*, potentially regulates spinal motor neuron maturity and function through pathways involving DSCAM and VEGF signaling [115,116]. Its deletion alters dendritic complexity, axonal growth, and electrophysiological properties, leading to impaired motor behaviors and reduced muscle strength in mice [117].

*Lhx1os*, with Isl1-Lhx3 complex, being a significant focus due to its impact on motor neuron homeostasis and function [118,119]. In mice, *Lhx1os* deletion induced motor impairment and a reduction in mature MNs, interacts with the ER-associated disulfide isomerase PDIA3 and modulates the unfolded protein response [9].

Axonally localized *Rmst* and *7H4* influence local translation and axon guidance [125], while soma-enriched *Lnc-MN1-3* may stabilize or regulate mRNA translation in post-mitotic MNs [126].

Collectively, lncRNAs act through epigenetic repression, transcriptional tuning, and spatially restricted RNA functions to ensure precise MN development. Their susceptibility to dysregulation provides a mechanistic link to motor neuron disease pathogenesis, a theme that will be explored in the following section on lncRNA-mediated ceRNA networks.

## 4. LncRNAs and Their Mediated ceRNA Networks in MNDs and Related Diseases

**Figure 2:**
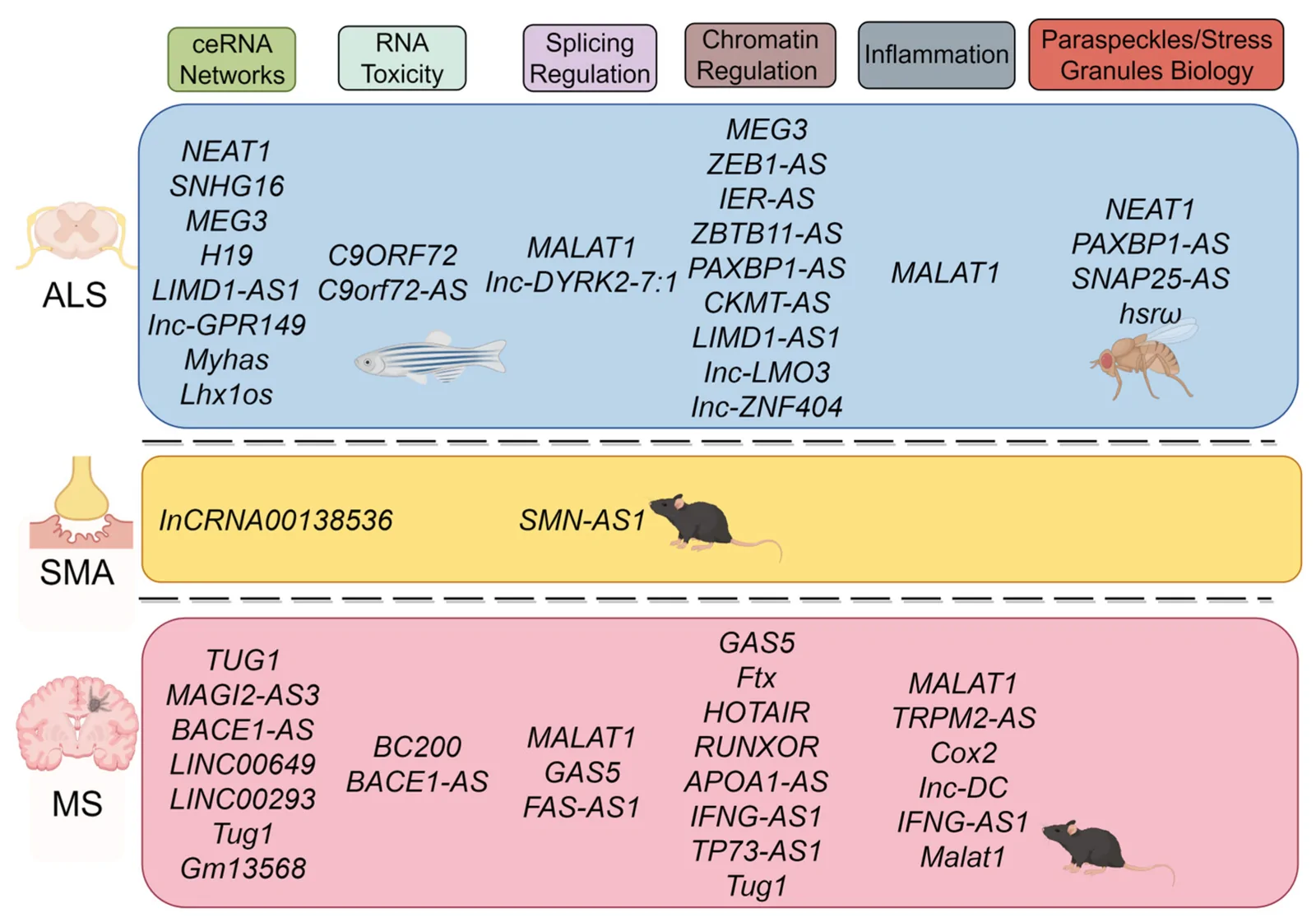
LncRNAs associated with different motor neuron diseases.

In MNDs, RNA metabolism and neuronal communication disruptions contribute to neurodegeneration. Evidence indicates that lncRNA-mediated ceRNA networks help regulate neuronal survival, axonal maintenance, and synaptic function. For instance, *CyCoNP* influences motor neuron physiology via the *miR-4492/NCAM1* axis [23]. Additionally, *lncIRS1* acts as a ceRNA, affecting the IGF1–PI3K–AKT pathway by sponging *miR-15* family members during muscle atrophy [132] (Figure 2).

### 4.1. ALS

ALS involves the progressive loss of MNs due to malfunctioning RBP like FUS, C9ORF72, TDP-43, hnRNPA1, ATXN2, ANG and TAF15 [133,134,135,136]. Compared with the normal 2–22 *GGGGCC* (*G_4_C_2_*) repeats in the intronic region of *C9ORF72* on Chr.9p, expansions of approximately 700–1600 copies are associated with ALS and FTD [137]. In addition, lncRNAs are increasingly recognized as important players in ALS, as TDP-43 has been shown to bind nuclear lncRNAs such as *MALAT1* and *NEAT1*, suggesting their involvement in TDP-43 mediated RNA dysregulation [138] (Table 4).

#### 4.1.1. Transcriptome Analysis of lncRNAs in ALS

In human ALS, *NEAT1* and *SNHG16* levels rise in the brainstem, while *MEG3* and *H19* increase in the frontal cortex, and *MALAT1* decreases there [139]. Similarly, SOD1^G93A^ mice show increased *H19*, *Myhas*, and *Neat1* in spinal cord, brainstem and frontal cortex across ALS symptomatic stages [139,140]. In ALS, *ZEB1-AS*, *IER-AS*, *ZBTB11-AS*, *PAXBP1-AS*, *SNAP25-AS* and *CKMT-AS* are up regulated in PBMC and spinal cord, with antisense lncRNAs *ZEB1-AS, ZBTB11-AS*, and *UBXN7-AS* linked to UMN degeneration [141]. Additionally, *EEF1A1P16/5/24* and *HNRNPA1P48* increased in ALS SEVs and LEVs [142], while *lnc-ABCA12-3:1* rises and *lnc-DYRK2-7:1* and *lnc-POTEM-4:7* fall in ALS PBMCs [143].

In mouse MNs derived from embryonic stem cells carrying *FUS-null* or *P517L* mutations, decreased expression of *Lhx1os* and *lncMN-1/2*, lncRNAs implicated in axon guidance and LMC specification [126]. Similarly, in patient-derived iPSCs carrying FUS R521H or P525L mutations, *lncLMO3*/*LMO3*, *lnc-ERMN/NR4A2 (Nurr1)*, and *lnc-ZNF404/ZNF404* were downregulated, whereas *lnc-GPR149*/*GPR149* and *CRACD* were upregulated in their differentiated MNs [144]. In PBMCs and lymphoblastoid cell lines (LCLs) from ALS patients, *LIMD1-AS1* is upregulated in *TARDBP* mutants [145].

#### 4.1.2. *C9ORF72-AS*

The expansion of the *GGGGCC* (*G_4_C_2_*) repeat in the 5′-UTR of *C9ORF72* induces neurotoxicity, which is a major genetic cause of ALS and FTD [81,137,146]. It forms G-quadruplex structures that trap RBP like TDP-43 and FUS, disrupting RNA functions [147], and leads to RAN translation, producing toxic DPR that impair cellular transport and protein balance [148,149]. In addition, the *G_4_C_2_* repeat expansion leads to reduced *C9ORF72* expression, resulting in loss-of-function toxicity by affecting autophagy and immune responses [150].

The frequency of *C9ORF72-AS* expression in patient neurons is reported to be higher than that of the sense transcripts [151,152,153]. The *C9ORF72-AS* is linked to RNA-mediated toxicity in peri-nucleolar regions of MNs and interneurons in the frontal cortex and spinal cord of C9-ALS/FTD patients [137,146,153,154,155]. Its toxicity is context-dependent, as shown by zebrafish models where it induces motor axonopathy [156], while in *Drosophila*, it sequesters RBP without causing neurodegeneration [152]. Unlike the sense strand, the *C9ORF72-AS* repeat sequences can form i-Motifs, hairpin structures, or double-helical structures with C:C mismatches [152,156,157,158,159]. Collectively, RNA-mediated toxicity, DPR protein toxicity, and *C9ORF72* loss-of-function act synergistically and intersect with shared ALS pathological features.

Above all, current evidence indicates that the pathogenic effects of *C9ORF72-AS* are primarily mediated through RNA toxicity, repeat-associated non-AUG translation, and RNA-binding protein sequestration rather than through a classical ceRNA mechanism.

**Table 4:** Evidence levels of lncRNA and circRNA associated ceRNA networks in MNDs.

Symbol	ceRNA Axis	Origin	Phenotypes	Mechanism	Evidence Level	Experimental Validation	Ref.
**lncRNAs**							
*CyCoNP*	*miR-4492/NCAM1* in *cis*	Human cell lines and iPSC	MN subtypes	Sustains the MN differentiation program in both neuroblastoma cells and hiPSC-derived motor neuron	A	Knockdown, miRNA inhibition, target validation	[23]
*lncRNA00138536*	*miR-34a/Spag5*	Human and mice	SMA	Regulates cell-cycle progression in human cell lines and SMA model	A	Experimental validation	[24]
*Ftx*	*miR-382-5p/NRG1*	Human blood	MS	Differential Diagnosis of MS and Neuromyelitis Optica	C	Expression profiling and biomarker analysis only, no direct ceRNA validation	[160]
*HOTAIR*	*miR-136-5p/AKT2*	Human and mice cell lines	MS	Regulates microglia switching to a pro-inflammatory M1-like phenotype in Sulfasalazine treatment	A	RIP + rescue	[161]
*lncIRS1*	*miR-15a/b/c*	Chicken tissue and cell line	MS	Regulate myoblast proliferation and differentiation in muscle and DF-1 cell	B	in vivo + in vitro	[132]
*MALAT1*	*miR-124-3p/Sgk1*	Mice EAE model and cell lines	MS	Promotes microglia activation in BV2 cell lines and neuronal apoptosis	A	Dual-luciferase assay, gain-/loss-of-function experiments, EAE validation, microglial activation analysis	[162]
*Tug1*	*miR-9-5p/NF-κB1*	Mice EAE model and cell lines	MS	Enhances pro-inflammatory responses via the *miR-9-5p/NF-κB1-p50* pathway	A	luciferase assay	[163]
*MAGI2-AS3*	*miR-374b-5p/PTEN*	Human blood	MS	Modulate the PTEN/AKT/IRF-3/IFN-β Axis (Predicted)	D	Bioinformatic prediction only	[164]
*NEAT1*	*miR-373-3p/-302c-3p/-372-3p*-APP	Human neural and non-neural tissue	sALS	NA (Predicted)	D	Bioinformatic prediction only	[165]
*XIST*	*miR-9-5p/30e-5p/671-5p*			NA (Predicted)	D	Bioinformatic prediction only	
*LINC00649*	*miR-1275–CD20*	Human PBMCs	MS	NA(Predicted)	D	Bioinformatic prediction only	[166]
**circRNA**							
*circSLC8A1*	*circDLC1*/*miR-335-3p*	human FUS^P525L^ MNs	MN and ALS pathology	Neuroprotection (Predicted)	D	prediction	[167]
*circDLC1*	*miR-217/HNRNPA3*			FUS toxicity (Predicted)	D	prediction	
*circCARHSP1*	*miR-24-3p/* *miR-345-5p*			Neurons and microglia viability (Predicted)	D	prediction	
*circ_0000002-*	*miR-302d-3p/373-3p-APP*	Human neural and non-neural tissue	sALS	NA (Predicted)	D	prediction	[162]
*circ_0063411*	*miR-647*	Human leukocytes	ALS	NA (Predicted)	D	prediction	[168]
*hsa_circ_0023919*	*miR-9*			NA (Predicted)	D	prediction	

Evidence levels were classified according to the strength of experimental support for ceRNA regulation. A: experimentally validated ceRNA regulation; B: functional evidence without complete ceRNA validation; C: transcriptomic or patient-associated evidence; D: computational prediction only.

#### 4.1.3. *NEAT1*

The *NEAT1/2* are highly expressed in early ALS stages of human, acting as scaffolds for TDP-43 and FUS/TLS in spinal MNs [169,170]. Disruption leads to protein mislocalization, stress granule formation, and abnormal paraspeckle dynamics [169,170]. The *NEAT1* cis-eQTL (rs10128627) is associated with earlier ALS onset [171], and the D169G mutation affects *NEAT1*’s role in TDP-43 phase separation and nuclear body formation [172]. The *NEAT1*–FUS/TLS–TDP-43 axis is crucial for nuclear homeostasis, and its dysregulation is a key pathogenic mechanism in ALS [173] (Table 4).

In hSOD1^G93A^ mice, *Neat1* knockout exacerbates TDP-43-mediated neurodegeneration and increased SOD1 aggregation [171,173,174,175]. Although initially neuroprotective, *NEAT1* dysregulation later exacerbates neuronal degeneration due to RBP mutations [8,141,170,175,176]. Additionally, *NEAT1* and *HSP70* collaboratively mediate *C9ORF72*-related neurotoxicity in humans [177]. Based on bioinformatic analysis, neural specific expressed *NEAT1*-*miR-373-3p/-302c-3p/-372-3p*-APP and *XIST*-*miR-9-5p/-30e-5p/-671-5p* ceRNA networks, may potentially regulate APP expression in ALS patients; however, direct experimental validation remains limited [165,178] (Table 4).

#### 4.1.4. *MALAT1*

*MALAT1*’s role in ALS has been linked to RNA metabolism and mitochondrial dysfunction across multiple CNS regions and cell types through interactions with TDP-43, *SAT1*, and several splicing-related factors, as well as the *Keap1/Nrf2* signaling pathway [138,179,180]. Additionally, *MALAT1*-regulated genes including *SYNRG*, *ITSN2*, *PICALM*, *AP3B1* and *AAK1* may contribute to ALS-related pathogenic processes [181].

#### 4.1.5. *SatIII*

In human, *SatIII* is a stress-induced lncRNA transcribed from satellite repeat regions and represents the orthologue of *Drosophila hsrω*. Notably, *SatIII* expression is elevated in FTD patient tissue as well as in cellular models of TDP-43 overexpression [182]. In *Drosophila*, increased *hsrω* aggravates TDP-43-induced neurotoxicity, whereas its suppression enhances autophagy-mediated TDP-43 clearance [182,183,184,185]. In addition, *hsrω* depletion causes cytoplasmic mis-localization and loss of nuclear dFUS function, and its knockdown in MNs impairs locomotion in flies by causing pre-synaptic terminal defects [183]. These findings support a role for *SatIII* in stress-responsive RNA regulation, although no direct ceRNA mechanism has yet been demonstrated.

#### 4.1.6. Other ceRNA Networks

In ALS-associated lymphoblastoid cell lines, reduced *ATXN2-AS* and its repeat expansion produce a neurotoxic RNA species [186]. Similar to *C9ORF72-AS*, *ATXN2-AS* contributes to ALS pathology through toxic RNA-mediated mechanisms rather than experimentally validated ceRNA regulation [186].

Several circRNAs have been identified in ALS through sequencing and bioinformatics. Such as, nervous system-specific expressed *Circ_0000002-miR-302d-3p/miR-373-3p-APP* may act as the potential biomarkers for the diagnosis of sporadic ALS [165] (Table 4). In human in vitro FUS^P525L^ MNs, several regulatory networks have been identified: *circSLC8A1*/*circDLC1*/*miR-335-3p* in neuroprotection, *circDLC1*/*miR-217*/*HNRNPA3* in FUS toxicity, *circCARHSP1*/*miR-24-3p/miR-345-5p* for neurons and microglia viability [167]. Additionally, circRNA *hsa_circ_0063411*/*miR-647*, *hsa_circ_0023919/miR-9*, and *hsa_circ_0088036* were up-regulated in peripheral leukocytes of ALS patients [168]. Computational analyses have identified a putative ceRNA network involving *MIR4435-2HG, AC005332.6, AC007389.1, AL139220.1*, and *AC004951.1* in ALS [187]. However, these interactions currently remain prediction-based and require further experimental validation.

### 4.2. SMA

SMA is caused by homozygous deletion or mutation of the *SMN1* gene, leading to reduced SMN protein levels and impaired snRNP assembly, RNA splicing, and axonal mRNA localization, with motor neurons being particularly affected [6,188,189]. Transcriptomic analyses of severe SMA patient models have suggested the presence of ceRNA regulatory networks involving *lncRNA00138536*, *miR-34a*, *circRNA007386*, and several cell-cycle–related genes [24].

Subsequent in vitro experiments demonstrated that SMN deficiency resulted in *miR-34a* dysregulation and its overexpression disrupted cell-cycle progression through regulation of targets such as *Spag5* and *Hjurp* (Table 4). However, the proposed ceRNA interactions remain largely based on network analyses and indirect functional evidence, and further mechanistic validation is required.

#### *SMN-AS1* 

The *SMN* loci generate two antisense lncRNAs, *SMN-AS1* in intron 1 and SMN-AS1* in intron 5 [190,191] (Figure 2), which overlap with Alu elements and may suppress SMN expression by recruiting the PRC2 to the SMN promoter or SMN body [11,192]. In SMA patient cells, antisense oligonucleotides (ASOs) knockdown of *SMN-AS1* increased *SMN2* transcription and exon 7 inclusion, raising SMN levels [11]. In severe SMA mice, *SMN-AS1*-targeting ASO modestly increased CNS SMN levels but lacked significant therapeutic effects [193]. However, combining it with an ISS-N1-targeting ASO led to noticeable therapeutic benefits [11].

Overall, current evidence for ceRNA regulation in SMA remains limited and is largely based on transcriptomic analyses. Additional mechanistic studies are required before these networks can be considered validated therapeutic targets.

### 4.3. Motor Dysfunction in Non-Motor Neuron Diseases: Exploratory Relevance of lncRNA-Mediated ceRNA Networks in MS

In MS, motor dysfunction is a key symptom, and axonal degeneration, neuronal injury, and chronic neuroinflammation contribute substantially to disease progression [194,195]. In addition, variants such as *HOTAIR*-rs4759314 [196], *GAS5*-rs2067079/rs55829688/rs6790 [197,198,199,200,201], *TRPM2-AS*-rs933151 [202] are associated with the increased risk of MS susceptibility and development [198,199]. Thus, this section includes studies on MS studies to offer insights into lncRNA and their-related ceRNA mechanisms (Table 4).

#### 4.3.1. Transcriptome Analysis of lncRNAs in MS

In human serum, the lncRNA *Ftx*/*miR-382-5p* regulatory pair has been suggested as a potential biomarker for distinguishing MS from neuromyelitis optical spectrum disorders [160]. LncRNAs (*MALAT1, GAS5, MEG3*, and *H19*) show consistent dysregulation in MS, whereas others (*THRIL, IFNG-AS1, HOTAIR*, and *TUG1*) exhibited context-dependent expression influenced by treatment, relapse status, or demographics [203].

*HIF1A-AS3*, *RUNXOR*, *AK018453*, *Gm13568, CTB-89H12.4* are associated with hypoxia- and inflammation-related pathways in progressive MS [202,204,205,206]. Subtype-specific signatures include increased *TUG1* and *MALAT1* in secondary progressive MS (SPMS), whereas altered expression of *LRRC75A-AS1*, *FAS-AS1*, *LINC00293*, *BC200, RP11-29G8.3*, *Cox2* and *lnc-DC* in relapsing-remitting MS (RRMS) [207,208,209,210,211,212]. Mechanistically, increased *APOA1-AS* and *IFNG-AS1* affect the RRMS activity and relapse rate through ApoA1/S1P signaling [213], and *BACE1-AS* is linked to cognitive issues in SPMS [211].

#### 4.3.2. *MALAT1*

*MALAT1* is highly expressed in the serum of SPMS [207], and validated as a ceRNA that sponges *miR-124-3p*, thereby derepressing *Sgk1* expression, activating IκBα/NF-κB signaling, and promoting microglial activation and neuronal apoptosis in EAE mice models [162].

Network-based analyses have identified *LINC00649*, *TP73-AS1*, and *MALAT1* as potential hub nodes within MS-associated ceRNA networks [166]. Among them, *LINC00649–miR-1275–CD20* axis may participate in the immunological and inflammatory pathogenesis of MS. In another study, *MALAT1* was shown to act as a ceRNA by sponging the miRNA *miR-338-3p*, indirectly inducing MSL2 expression in MG [214]. However, these findings remain largely computational and await experimental validation.

#### 4.3.3. *HOTAIR*

In sulfasalazine (SF) treated microglia, *HOTAIR* is down-regulated but *miR-136-5p* is up-regulated, and thus inactivated *AKT2-NF-κB* pathway [161] (Table 4). Importantly, lncRNA *HOTAIR* overexpression reverses the increased *miR-136-5p* expression by SF and thereby attenuated the inhibition of *AKT2*-mediated *NF-κB* activation. Mimic of *miR-136-5p* inhibited cuprizone-induced activation of *AKT2-NF-κB* in the microglia. Above all, sulfasalazine suppresses M1 microglial polarization partly through modulation of *HOTAIR miR-136-5p*/*AKT2–NF-κB* axis, thereby promoting remyelination [161].

#### 4.3.4. *TUG1*

The expression of *TUG1* was found to be upregulated in serum samples obtained from MS patients [215]. In EAE mice, down-regulated *TUG1* improved mice behavior, reduced granulocyte-macrophage colony stimulating factor (GM-CSF) level, decreased the levels of TNF-α, IFN-γ, IL-6/17, and increased IL-10 [163]. Additionally, *TUG1* expression was negatively correlated with *miR-9-5p*, while positively correlated with *NF-κB1/p50*. Knockdown of *TUG1* or enforced expression of *miR-9-5p* inhibited LPS-induced inflammation in BV2 cells, while these effects were abolished by inhibition of *miR-9-5p*. Above all, down-regulation of *TUG1* attenuated MS through inhibition of inflammation by sponging *miR-9-5p* via targeting *NF-κB1/p50* (Table 4).

#### 4.3.5. *GAS5*

As an epigenetic regulator, *GAS5* was highly expressed in the MS patients, and its variants rs2067079/rs55829688/rs6790 in brain and immune system are associated with the increased risk of relapsing-remitting and secondary-progressive MS [197,198,199,200,201]. In a lysolecithin-induced demyelination model of mice, interference with *GAS5* in transplanted microglia attenuates the progression of experimental autoimmune encephalomyelitis (EAE) and promotes remyelination [216]. Further functional studies in cultured microglia and OPCs demonstrate that GAS5 suppresses transcription of TRF4, by recruiting the polycomb repressive complex 2 (PRC2), thereby inhibiting M2 polarization.

### 4.4. Therapeutic Implications of lncRNA-Mediated ceRNA Networks in Motor Neuron Diseases

#### 4.4.1. Antisense Oligonucleotides (ASOs)

The growing understanding of lncRNA-mediated regulation has opened new avenues for targeted therapies in MNDs [7,217], which leading to RNA degradation, blocking RNA-protein interactions, or altering pre-mRNA splicing [218]. The success of nusinersen (Spinraza) in treating spinal muscular atrophy (SMA), through the enhancement of SMN protein expression and improvement of motor function, underscores the potential of RNA-targeting strategies. Antisense oligonucleotides (ASOs) targeting disease-associated lncRNAs, such as SMN-AS1 and C9ORF72-AS, hold promise for restoring gene expression or mitigating toxic RNA accumulation [11,219] (Table 5). Nonetheless, despite these encouraging developments, ASO therapy continues to encounter several challenges, including the necessity for repeated intrathecal administration, restricted tissue distribution, and potential off-target effects.

#### 4.4.2. Small Interfering RNAs (siRNAs)

SiRNAs silence gene expression by degrading target mRNAs through the RNA interference pathway, which are more effective than ASOs in reducing overexpressed disease-related transcripts. Research indicates that siRNA can suppress pathogenic lncRNAs, potentially reducing neuroinflammation, protein aggregation, and neuronal apoptosis [220]. SiRNAs can also target specific components of lncRNA-mediated ceRNA networks. However, delivering siRNAs to motor neurons is challenging due to their poor stability in vivo and limited ability to cross the blood-brain barrier [217,221] (Table 5).

#### 4.4.3. Gapmer ASOs

Gapmer ASOs are modified antisense oligonucleotides with a central DNA region that attract RNase H to degrade nuclear lncRNAs, making them ideal for targeting nucleus-localized lncRNAs like *NEAT1*, *MALAT1*, and *HOTAIR* [218]. They are more potent than traditional ASOs for degrading nuclear transcripts, but further optimization of their chemical modifications and delivery systems is needed to enhance efficacy and reduce toxicity [217,218] (Table 5).

#### 4.4.4. CRISPR-Based Genome and RNA Editing

Genome-editing technologies provide another promising strategy for manipulating lncRNA function. CRISPR/Cas9 can permanently disrupt pathogenic lncRNA loci or modify regulatory elements controlling lncRNA transcription. More recently, CRISPR interference and CRISPR activation have enabled reversible regulation of lncRNA expression without altering genomic DNA. RNA-editing technologies, including CRISPR/Cas13 and adenosine deaminase acting on RNA (ADAR)-based systems, further permit transient correction of disease-associated RNA transcripts while avoiding permanent genome modification. These approaches may provide higher specificity for correcting pathogenic RNA dysregulation in ALS and SMA [222]. However, efficient delivery, editing efficiency, and long-term safety remain important challenges before clinical translation [217,222] (Table 5).

#### 4.4.5. Delivery Strategies for RNA Therapeutics

Efficient delivery is a major challenge for RNA-based therapies for motor neuron diseases due to the need to cross the blood-brain barrier (BBB) [217]. Intrathecal injection is the most common method for administering ASO therapy, as seen with nusinersen in SMA patients. Viral vectors like adeno-associated virus (AAV) offer effective gene delivery and long-term expression but can trigger immune responses and have limited cargo capacity [221]. Researchers are exploring lipid nanoparticles, exosomes, and other nanocarriers to enhance delivery efficiency and reduce toxicity [217,218]. Optimizing delivery methods is crucial for effectively targeting motor neurons with minimal side effects (Table 5).

#### 4.4.6. Safety Considerations and Future Perspectives

Although RNA-targeted therapies show promise, safety concerns persist, including off-target effects that may alter gene expression and immune activation from synthetic oligonucleotides or viral vectors, potentially affecting efficacy. Long-term use might lead to toxicity or reduced compliance [217]. Many lncRNA-mediated ceRNA networks lack thorough validation, relying on transcriptomic data or predictions [7]. Future development should focus on experimentally validated networks with clear pathogenic relevance. Using single-cell and spatial transcriptomics, along with precise genome-editing, will help identify reliable therapeutic targets and speed up the clinical application of lncRNA-based therapies [7,217,222] (Table 5).

## 5. Conclusions

LncRNAs play a crucial role in motor neuron development, function, and disease progression. They are involved in various pathogenic processes in MNDs such as RNA metabolism, neuroinflammation, synaptic dysfunction, and neuronal survival. The ceRNA regulation mediated by lncRNAs is a promising research area, although the strength of evidence varies across studies. Many interactions are still based on predictions or correlations and need experimental validation. Future research combining single-cell and spatial transcriptomics with functional studies is essential to understand lncRNA functions and identify biomarkers and therapeutic targets for MNDs.

## 6. Limitation

This review symmetrically reviewed current literature on lncRNAs in MN development and related diseases, highlighting their involvement in ceRNA network in different tissues and species. However, several limitations remain unresolved. Firstly, most lncRNA-associated ceRNA networks in MNDs rely on transcriptomic analyses and bioinformatic predictions, with limited direct validation. Secondly, there is significant heterogeneity in disease models, sample sources, and analytical methods, which affects consistency and comparability. Thirdly, the lack of standardized criteria for identifying and validating ceRNA interactions complicates distinguishing true mechanisms from indirect associations.

This review summarizes the limited database and validated ceRNA network, emphasizing the need for advanced computational tools and databases. Future research should incorporate multi-omics in scRNA-seq and scStereo-seq, functional experiments, and patient-derived models to validate ceRNA networks and understand their role in motor neuron development and disease.

## Figures and Tables

**Table 1:** Spinal Motor Columns: Segmental Distribution and Functional Targets.

Molecular Feature	Disease	Functional/Pathological Note	Ref.
**MMC**			
Wnt4/5 signaling	NA	Reinforces MMC fate; affects trunk connectivity	[37]
Isl2		Organizes MMC and LMC into motor pools	[38]
*BASP-AS1/ISL1-DT*		Highly expressed in MMC	[14]
FGF	ALS	Autocrine/paracrine survival factor	[39]
		Astrocytic FGF-2 affects motor neurons in ALS mice model	[40]
Lhx3/4		Maintains axial MN identity	[37]
		iPSC-MNs selectively targeted muscles innervated by Lhx3 MNs	[41,42]
Shh signaling		Early specification of MMC fate; axial connectivity	[43,44]
		Play the neuroprotective role via PI3K/AKT in ALS mice	[45]
RA		generate MMC-like neurons from stem cells	[46]
**LMC**			
EphrinB2	NA	regulates LMC boundary and axon pathfinding	[47]
*Bmpr1a*		the misrouting of LMCm axons	[30]
SC1 (BEN/DM-GRASP/neurolin)		Promotes axon fasciculation and columnar organization	[48]
*Hoxc6*		RA-regulated; ensures proper LMC specification	[43,49]
*LINC02381/FOXD3-AS1*		Highly expressed in LMC	[14]
*miR-17~92*	ALS	Cause the degeneration of LMC in ALS	[50]
**HMC**			
Foxp1	NA	Distinguishes HMC from LMC neurons	[51,52]
*SNHG14/LINC00632*		Highly expressed in HMC	[14]
ISL1-LHX3 complex		Establishes HMC identity	[53]
*Olig2^+^* progenitors	ALS	Developmental origin of HMC neurons	[54]
		Pathogenesis of Amyotrophic Lateral Sclerosis	[55]
Kdm6b	SMA	Promotes HMC and MMC fate Suppresses LMC/PGC programs	[56]
		Flunarizine-Mediated Neuroprotection in SMA	[57]

**Table 2:** Structural and functional characteristics of somatic motor neuron subtypes.

Type	Target Fibers	Diseases	Pathological Features	Ref.
**α-MN**	extrafusal	ALS	Selectively α-MN degeneration	[63,64,65]
**α-MN**	extrafusal	SMA	α-MN reduction	[66,67]
**α-MN**	extrafusal	MS	α-motor neuron output abnormalities	[68]
**β-MN**	extrafusal/partial intrafusal	PLS	UMN degeneration, cortical atrophy, oxidative stress/lipid peroxidation	[69,70,71]
**γ-MN**	Intrafusal	ALS	Cortical hyperexcitability precedes LMN loss; GDNF loss cause selective γ-MN degeneration	[72,73,74,75]

**Table 3:** Key lncRNAs Involved in Motor Neuron Development.

lncRNA	Locus	Key Mechanism & Outcome
**Early patterning**		
*Meg3*	14q32.2	1. rostro caudal MN identity by recruiting the PRC2:Jarid2 complex [100,101,102] 2. MN subtype specification by Ezh2–Jarid2 interaction [100]
*Pnky*	6q16.1	1. NSC migration by SHH via PTBP1 [103,104] 2. NSC differentiation by *Pnky*–PTBP1 complex [45,105], 3. NSC proliferation by Wnt/β-catenin pathway in ALS and SMA [106]
**Subtype specification**		
*MNX1-AS1*	7q36.3	1. specific motoneuron subtypes by *MNX1* [8,107]
*CAT7*	7q21.12 (zebrafish)	1. early MN subtype activation by *MNX1* [108,109,110] 2. regional identity by PRC1 component (*Ring1*) [111]
*lncrps25*	10:44,296,618–-44,301,820 (zebrafish)	1. MN formation and locomotion by encoding micropeptide [112,113] 2. MN phenotype by *Olig2* expression [113]
*lnc158*	4 C3 (mouse)	1. oligodendrocyte differentiation by binding *NFIB* 3′UTR [114].
**Neuronal maturity and apoptosis**		
*Litchi*	14 B (mouse)	1. orchestrate spinal circuit by DSCAM and VEGF [115,116] 2. regulates dendritic complexity, axonal growth [117]
*Lhx1os*	17q12	1. maintain MN homeostasis [118,119] 2. motor dysfunction and a reduction in the number of mature MNs [9]
*Malat1*	11q13.1	1. synaptic organization [120,121] 2. neuronal apoptosis via *miR-199a-5p/PRDM5* and miR-124 [122,123] 3. neural recovery by microglial M1 polarization and neuroinflammation [124]
*Rmst/7H4*	12q23.1	1. axon guidance in MNs [125]
*Lnc-MN1-3*	22q12.1	1. stabilize mature MNs [126]

**Table 5:** Comparison of therapeutic strategies targeting lncRNA-mediated regulatory networks in motor neuron diseases.

Strategy	Mechanism	Advantages	Limitations	Clinical Status
**ASOs**	RNase H-mediated degradation or splice modulation	High specificity; clinically validated (e.g., Spinraza for SMA); suitable for nuclear lncRNAs	Repeated intrathecal administration; off-target effects; limited tissue distribution	Approved for SMA; emerging in ALS
**siRNA**	RNA interference-mediated degradation of target RNA	Efficient knockdown of cytoplasmic transcripts; sequence-specific	Poor BBB penetration; delivery challenges; limited stability	Preclinical
**Gapmers**	RNase H-mediated degradation of nuclear lncRNAs	Highly effective for nuclear lncRNAs; strong gene silencing	Potential hepatotoxicity and off-target toxicity; delivery optimization required	Preclinical
**CRISPR/Cas9**	Permanent genome editing	Long-lasting therapeutic effects; precise genome modification	Ethical concerns; off-target genome editing; delivery difficulties	Experimental
**CRISPR/Cas13/RNA editing**	Programmable RNA editing without DNA modification	Reversible; reduced genomic risk; transcript-specific regulation	Editing efficiency and delivery require optimization	Experimental
**AAV-mediated delivery**	Viral vector-mediated gene delivery	High transduction efficiency; long-term expression	Immune responses; limited packaging capacity	Clinical trials/Preclinical
**Lipid nanoparticles/Exosomes**	Non-viral RNA delivery	Lower immunogenicity; repeat administration possible	Targeting efficiency and CNS delivery remain challenging	Preclinical

## Data Availability

No new data were created or analyzed in this study. Data sharing is not applicable to this article.
